# Time-Restricted Feeding Potentiates the Ability of *Lacticaseibacillus casei* to Enrich the Retina in Omega-3 Fatty Acids

**DOI:** 10.14336/AD.2023.0324

**Published:** 2023-12-01

**Authors:** Pierre Lapaquette, Anne-Sophie Boucard, Florian Chain, Stéphane Grégoire, Luis G. Bermúdez-Humarán, Niyazi Acar, Marie-Agnès Bringer

**Affiliations:** ^1^Univ. Bourgogne Franche-Comté, Agrosup Dijon, UMR PAM A 02.102, Dijon, France.; ^2^INRAE, AgroParisTech, Micalis Institute, Université Paris-Saclay, 78350, Jouy-en-Josas, France.; ^3^Centre des Sciences du Goût et de l'Alimentation, CNRS, INRAE, Institut Agro, Université de Bourgogne Franche-Comté, Dijon, France.

**To the Editor**,

Current treatments for age-related macular degeneration (AMD) —the primary cause of visual impairment in western countries in people over 50 [1]— are limited to a single form of the disease and cannot always avoid severe visual loss. Among emerging preventive strategies, omega-3 (n-3) polyunsaturated fatty acids (PUFAs) are drawing interest as they promote normal retinal structure and function, reduce incidence, and slow progression of AMD, consistently with their abundance in retinal neurons. Evidence has also accumulated suggesting the influence of the gut microbiota in AMD pathophysiology [2]. In this work, we investigated whether strategies targeting the gut microbiota could be effective in modulating retinal PUFA content.

Changes in the gut microbiota of AMD patients have been reported and several experimental studies suggest the existence of a gut-retina axis, in which gut microbes would influence AMD-associated mechanisms (inflammation, pathological vascularization and neurodegeneration) and modulate retina physiology, including its lipid composition [2]. Approaches targeting the gut microbiota such as probiotic-based strategies and intermittent fasting —a dietary intervention that shapes the gut microbiota to benefit health— have been shown to influence lipid metabolism and to protect the retina against harmful conditions [2, 3]. In the urgency context to find new strategies to prevent or delay the onset and progression of AMD, we evaluated the ability of supplementation with the probiotic *Lacticaseibacillus casei* (*L. casei*) strain BL23 and time-restricted feeding intervention to modulate the PUFA content in the mouse retina.

To do so, C57BL/6JrJ mice were randomly distributed into two groups: one group had a permanent access to food (*ad libitum* group) whereas the other had a time-restricted access to food (isocaloric twice-a-day feeding (ITAD) group, [4]) (Supplementary Fig. 1A and Supplementary data). Half of mice in each group were supplemented with *L. casei* BL23 by daily oral gavage (Supplementary Fig. 1B and Supplementary data). To evaluate the impact of these treatments on lipids, amounts of fatty acid methyl esters (FAMEs, which are derivatives of fatty acids carried by diacylglycerophospholipids and plasmalogens) and dimethylacetals (DMAs, which are derivatives of fatty alcohols on plasmalogens) were determined by gas chromatography (Supplementary data). For microbiota analysis, 16S rRNA gene sequencing was done on fecal material using the Illumina MiSeq technology (Supplementary data).

The analysis of fecal microbiota composition revealed that alpha diversity (Shannon and inverse Simpson indices) was significantly increased in mice supplemented with *L. casei* compared to ITAD fed mice (Fig. 1A). A trend to a decreased diversity (Shannon indice) was observed in gut microbiota of ITAD-fed mice compared to *ad libitum*-fed mice (Fig. 1A). Linear discriminant analysis effect size (LEfSe) approach showed that the feeding rhythm and *L. casei* supplementation were associated with specific changes in gut microbiota, including overrepresentation of: (i) *Prevotellaceae* —a family including producers of short chain fatty acids that are metabolites regulating of host lipid metabolism and health [5]— in *L. casei*-supplemented mice; (ii) *Lactobacillaceae* —a family including species having probiotic potential [6]— in ITAD-fed mice; and (iii) *Eggerthellaceae* —a family with members involved in the production of health-promoting metabolites (urolithins) [7]— in ITAD-fed mice supplemented with *L. casei* (Fig. 1B and 1C).

**Figure 1.**
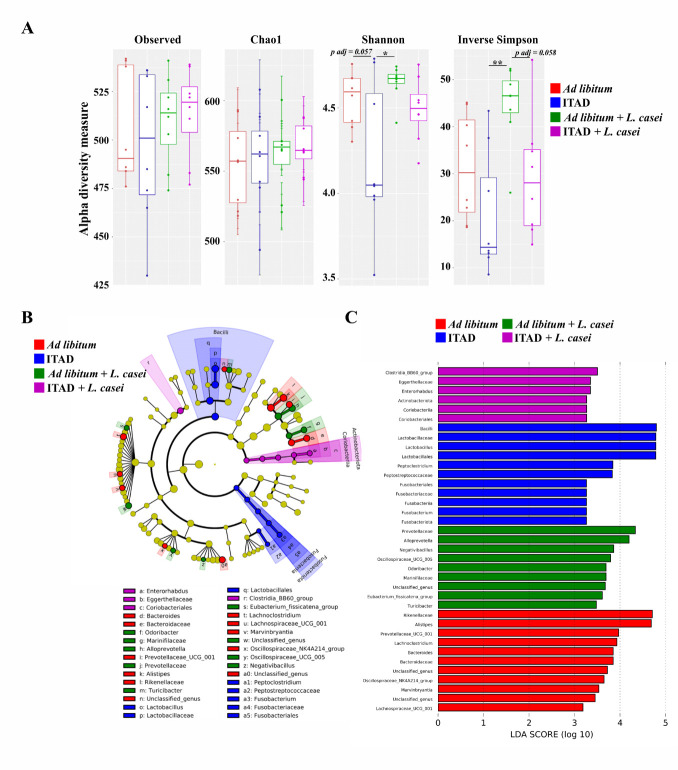
**Effect of ITAD feeding and *Lacticaseibacillus casei* BL23 supplementation on gut microbiota**. (**A**) Four indices (observed, Chao1, Shannon and inverse Simpson) of alpha diversity were analyzed. (**B** and **C**) LEfSe analysis was performed to identify significantly altered taxa in one group compared to another. (**B**) LEfSe cladogram. (**C**) LDA scores. Alpha value for the factorial Kruskal-Wallis test among classes: 0.05. Threshold on the logarithmic LDA score for discriminative features: 2.0. n=8 per group. * *p*<0.05, ** *p*<0.01.

The combination of ITAD feeding with *L. casei* supplementation deeply impacted lipids in the retina by significantly enriching its PUFA content (Fig. 2). The high levels of PUFAs were balanced by decreased abundances of saturated and monounsaturated fatty acids (Fig. 2A and 2B). At the species level, this strategy significantly modified the relative abundance of 17/24 lipid species *versus* 4/24 in ITAD-fed mice and 7/24 *L. casei*-supplemented mice compared to *ad libitum* fed-mice (Fig. 2B). Particularly, the abundance of several n-6 PUFAs (C20:3n-6, C20:4n-6 (arachidonic acid, ARA) and C22:4n-6) and n-3 PUFAs (C22:5n-3 and C22:6n-3 (DHA)) was significantly increased in the retina of ITAD-fed and *L. casei*-supplemented mice (Fig. 2B). Moreover, ITAD feeding combined with *L. casei* supplementation significantly increased the plasmalogens content in the retina as shown by the decrease in total FAMEs (fatty acid methyl esters) / total DMAs (dimethylacetals) ratio and the increased abundance of 2 DMA species (Fig. 2H and 2I). Plasmalogens are glycerophospholipids that concentrate PUFAs such as ARA and DHA. These PUFAs may be involved in the regulation of retinal vascular development after being released from plasmalogens [8].

**Figure 2.**
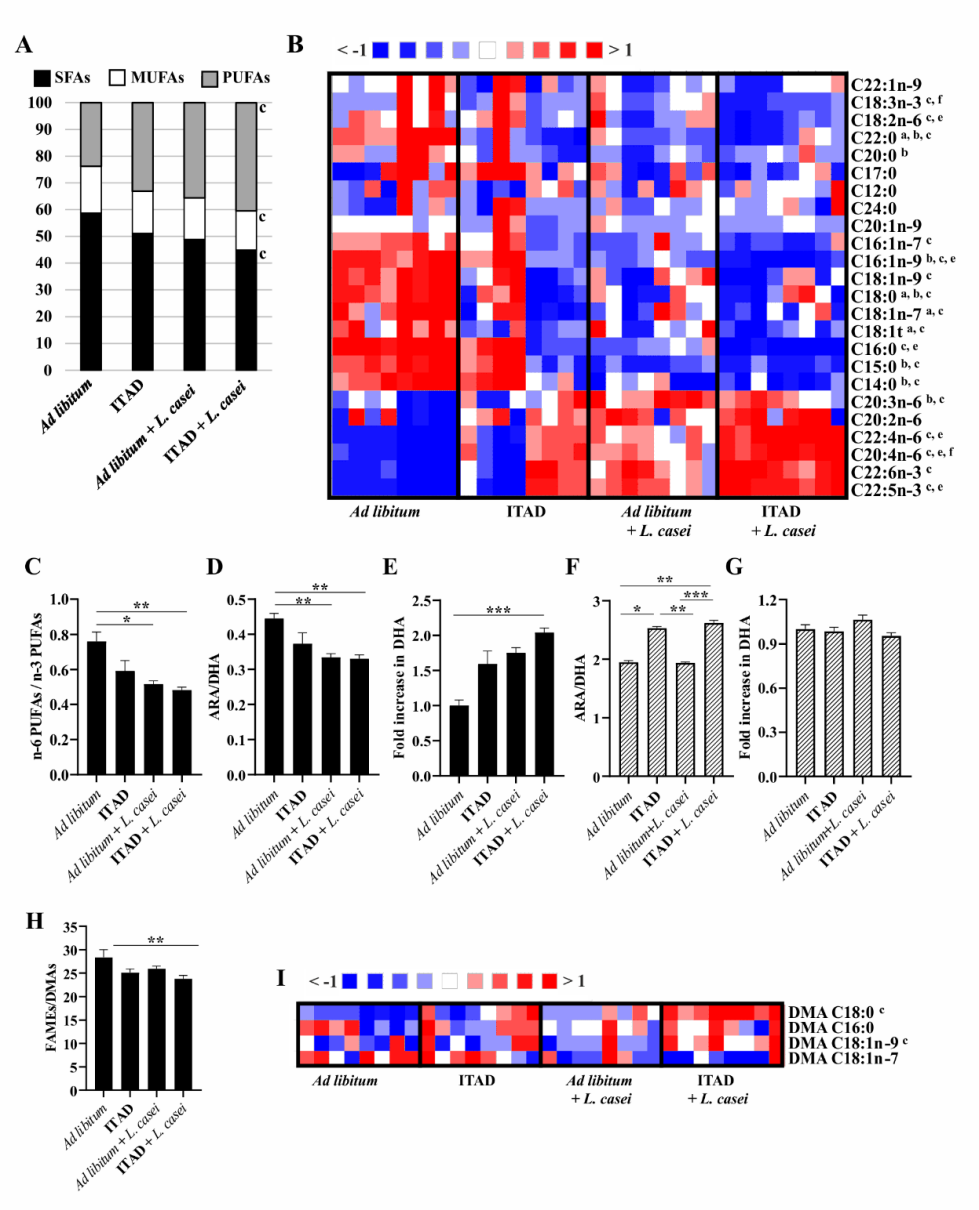
**Effect of ITAD feeding and *Lacticaseibacillus casei* BL23 supplementation on retinal and liver lipid contents in mice**. (**A**) Retinal abundance of total saturated fatty acids (SFAs), total monounsaturated fatty acids (MUFAs) and total polyunsaturated fatty acids (PUFAs) relative to total fatty methyl esters (FAMEs; 100%). (**B**) Heat map showing the retinal abundance of individual fatty acids relative to total FAMEs (100%). Each column corresponds to the retina of one mouse. (**C**) Ratio of total n-6 PUFAs/total n-3 PUFAs in retinas. (**D**) Ratio of arachidonic acid (ARA, C20:4n-6)/docosahexaenoic acid (DHA, C22:6n-3) in retinas. (**E**) Fold increase in retinal DHA. The retinal amount of DHA of *ad libitum*-fed mice was defined as 1.0. (**F**) Ratio of ARA/DHA in livers. (**G**) Fold increase in liver DHA. The liver amount of DHA of *ad libitum*-fed mice was defined as 1.0. (**H**) Ratio of total FAMEs/total dimethylacetals (DMAs) in retinas. (**I**) Heat map showing the retinal abundance of individual DMA species relative to total FAMEs + total DMAs. Each column corresponds to the retina of one individual. Kruskal-Wallis test with Dunn’s test for multiple comparisons. Significance: ^a^ *ad libitum versus* ITAD, ^b^ *ad libitum versus ad libitum* + *L. casei*, ^c^ *ad libitum versus* ITAD + *L. casei*, ^d^ ITAD *versus ad libitum* + *L. casei*, ^e^ ITAD *versus* ITAD + *L. casei*, and ^f^ *ad libitum* + *L. casei versus* ITAD + *L. casei*. * *p*<0.05, ** *p*<0.01, *** *p*<0.001. (C-H) Data are presented as mean ± SEM. n=8 per group.

An excessive amount of n-6 PUFAs and a high n-6/n-3 ratio are promoting factors for many diseases including AMD [1]. We observed that *L. casei* supplementation alone or in combination with ITAD feeding significantly reduced the n-6/n-3 ratio in mouse retinas as well as that of ARA/DHA, which are the most abundant retinal n-6 and n-3 PUFAs (Fig. 2C and 2D). This phenotype is likely to be the consequence of the strong enrichment in DHA whose abundance was doubled in ITAD-fed mice supplemented with *L. casei* (Fig. 2E). Interestingly, this enrichment was higher than those reported in rodents supplemented with dietary DHA and/or its precursor, eicosapentaenoic acid (EPA) [9]. It may result from endogenous synthesis since the ARA/DHA ratio was increased and the amount of DHA was unchanged in the liver of ITAD-fed mice supplemented with *L. casei* (Fig. 2F and 2G).

In conclusion, this study provides evidence that time-restricted feeding combined to probiotics is a promising strategy to modulate retinal DHA content, and thus to sustain retinal homeostasis during aging. Further investigations are needed to decipher the molecular mechanisms involved in the microbiota-retina dialogue, as well as to evaluate the efficacy of such a strategy in protecting the retina against pathophysiological mechanisms related to AMD.

## Supplementary Materials

The Supplementary data can be found online at: www.aginganddisease.org/EN/10.14336/AD.2023.0324.



## Data Availability

For microbiota, the raw datasets are available in the Sequence Read Archive (SRA) database system under project accession number PRJNA927267. All other data supporting the findings reported herein are available on reasonable request to the corresponding author.
