# Supplementary material for: Time-Restricted Feeding Potentiates the Ability of Lacticaseibacillus casei to Enrich the Retina in Omega-3 Fatty Acids
Source: Aging Dis. 2023 Dec 1;14(6):1945–9. doi: 10.14336/AD.2023.0324 (PMC10676782; doi:10.14336/AD.2023.0324)
Supplement: Supplementary file 1 [file AD-14-6-1945-s.pdf]

SUPPLEMENTARY DATA

Time-Restricted Feeding Potentiates the Ability of *Lacticaseibacillus casei* to Enrich the Retina in Omega-3 Fatty Acids

Pierre Lapaquette, Anne-Sophie Boucard, Florian Chain, Stéphane Grégoire, Luis G. Bermúdez-Humarán, Niyazi Acar, Marie-Agnès Bringer

© 2023. Lapaquette P et al. Published online at http://www.aginganddisease.org/EN/10.14336/AD.2023.0324

SUPPLEMENTARY DATA

MATERIALS AND METHODS

Mice and experimental conditions:

Male C57BL/6JrJ mice (7 weeks old; Janvier, France) were maintained at UE IERP (INRAE, Jouy-en-Josas, France) under specific pathogen-free conditions (12h/12h light/dark, water *ad libitum*). The experiments were approved by the local committee and performed in accordance with the European Community standards for animal care (authorization number 16744-201807061805486 v2).

Mice were randomly distributed into two groups (n=16/group): one group of mice had unrestricted access to food throughout the day and night (*ad libitum* group) and the other group was exposed to a time-restricted feeding protocol (isocaloric twice-a-day feeding (ITAD) group; [1]) (Supplementary Fig. 1A). In the ITAD group, mice had access to food only from 8 am to 10 am and from 4 pm to 6 pm. These two feeding protocols (*ad libitum versus* ITAD) were applied for 44 days.

During the last 25 days of the experiment, half of the mice (n=8/group) was supplemented with probiotic by daily oral gavage with $1x10^9$ colony-forming units (CFU) of *L. casei* BL23 in 200 µL of PBS-glycerol (Supplementary Fig. 1B). The other half of mice received by daily oral gavage 200 µL of PBS-glycerol alone. At the end of the assay, retinas and livers were collected on euthanized mice and snap-frozen in liquid nitrogen. Samples were kept in -80°C freezer until use.

FAME and DMA determination:

Hepatic and retinal total lipids were extracted according to the Folch Procedure [2]. They were transmethylated using boron trifluoride in methanol [3]. The fatty acid methyl esters (FAMEs; formed from fatty acids of diacylglycerophospholipids and plasmalogens) and dimethylacetals (DMAs; formed from fatty alcohols of plasmalogens) were further analyzed by gas chromatography coupled to flame ionization detection (GC-FID). FAMEs and DMAs were extracted with hexane and analyzed on a GC Trace 1310 (Thermo Scientific, Les Ulis, France) gas chromatograph (Palo Alto, CA, USA) using a CPSIL-88 column (100 m × 0.25 mm i.d., film thickness 0.20 µm; Varian, Les Ulis, France) equipped with a flame ionization detector. Hydrogen was used as carrier gas (inlet pressure 210 kPa). The oven temperature was held at 60 °C for 5min, increased to 165 °C at 15 °C/min and held for 1min, and then to 225 °C at 2 °C/min and finally held at 225 °C for 17min. The injector and the detector were maintained at 250 °C. FAMEs and DMAs were identified by comparison with commercial and synthetic standards. The data were processed using the ChromQuest software (Thermo Scientific).

Microbiota analyses:

DNA extraction, library preparation and Illumina sequencing were performed on the "@BRIDGe platform" (INRAe, Jouy-en-Josas, France)

DNA extraction. A modified version of the protocol by Godon *et al* was used for DNA extraction [4]. For each mouse, 200 mg of frozen fecal sample were resuspended in 250 µL of 4 M guanidine thiocyanate-0.1 M Tris (pH 7.5), 40 µL of 10% N-lauroyl sarcosine (pH 8.0) and 500 µL of 5% N-lauroyl sarcosine (pH 8.0), and incubated 1 h at 70°C. One volume (750 µL) of 0.1 mm diameter silica beads (Sigma) was added, and tubes were shaken 10 min at maximum speed of Vibrobroyeur MM200 (Retsch, Germany). Tubes were vortexed and centrifuged 5 min at 14,000 rpm at 4°C. Supernatants were incubated with 30 µL of Proteinase K (Chemagic STARDNA BTS kit, Perkin Elmer, USA) 10 min at 70°C at 250 rpm in Multi-Therm (Benchmark Scientific, USA) and 5 min at 95°C. Tubes were centrifuged 5 min at 14,000 rpm at 4°C and supernatants were transferred in a deepwell on the nucleic acid workstation Chemagic STAR (Hamilton, Perkin Elmer, USA). The extraction protocol was performed with Chemagic STAR DNA BTS kit (Perkin Elmer, USA) according to manufacturer instructions.

Library preparation. The V3-V4 hyper-variable regions of 16S rDNA gene were amplified from DNA extracts using universal primers PCR1F_343 (CTTTCCCTACACGACGCTCTTCCGATCTACGGRAGGCAGCAG, partial P5 adapter–primer) and PCR1R_784 (GGAGTTCAGACGTGTGCTCT-TCCGATCTTACCAGGGTATCTAATCCT, partial P7 adapter–primer) [5]. PCR was performed using 2 U Taq DNA Polymerase, Taq DNA polymerase buffer (MTP Taq DNA Polymerase, Sigma-Aldrich, USA), 10 nmol of dNTP mixture (Sigma-Aldrich, USA), 15 nmol of each primer (Eurofins, Luxembourg) and Nuclease-free water (Qiagen, Germany) in a final volume of 50 µL. The PCR reaction was performed with T100 Thermal cycler (Biorad, USA) as follows: an initial denaturation step (94°C for 10 min) was followed by 30 cycles of amplification (94°C for 1 min, 68°C for 1 min and 72°C for 1 min) and a final

© 2023. Lapaquette P et al. Published online at http://www.aginganddisease.org/EN/10.14336/AD.2023.0324

elongation step at 72°C for 10 min. Amplicons were purified using magnetic beads CleanPCR (Clean NA, GC biotech B.V., Netherlands) in a 96 well format. Concentration of purified amplicons was controlled using a Nanodrop spectrophotometer (Thermo Scientific, USA) and a subset of amplicons size was controlled on a Fragment Analyzer (AATI, USA) with the reagent kit ADNdb 910 (35-1,500 bp). Sample multiplexing was performed by adding tailor-made 6 bp unique indexes during the second PCR step at the same time as the second part of the P5F/P7R adapters to obtain primers PCR2_P5F (AATGATACGGCGACCACCGAGATCTACACT-CTTTCCCTACACGAC, partial P5 adapter–primer targeting primer 1F) and PCR2_P7R (CAAGCAGAAGACGGCATACGAGAT-NNNNNN-GTGACT-GGAGTTCAGACGTGT, partial P7 adapter including index–primer targeting primer 1R) [6]. This second PCR step was performed on 50–200 ng purified amplicons using 2.5 U DNA free Taq DNA Polymerase, Taq DNA polymerase buffer, 10 nmol of dNTP mixture (Sigma-Aldrich, USA), 25 nmol of each primer (Eurofins, Luxembourg) and Nuclease-free water (Qiagen, Germany) in a final volume of 50 µL. The PCR reaction included an initial denaturation step (94°C for 10 min), 12 cycles of amplification (94°C for 1 min, 65°C for 1 min and 72°C for 1 min) and a final elongation step at 72°C for 10 min. Amplicons were purified as described for the first PCR reaction. Concentration of purified amplicons was measured using a Nanodrop spectrophotometer (Thermo Scientific, USA) and quality of a subset of amplicons (12 samples per sequencing run) was controlled on a Fragment Analyzer (AATI, USA) with the reagent kit ADNdb 910 (35-1,500 bp). Libraries were pooled with equal amounts in order to generate equivalent number of raw reads for each library. DNA concentration of the pool was quantified on a Qubit Fluorometer (Thermofisher Scientific, USA). The pool at a final concentration between 5 and 20 nM was used for sequencing.

Illumina sequencing. The pool was denatured with 0.1N NaOH, diluted to 7 pM with 15% PhiX Control v3 (Illumina, USA) and 600 µL were then loaded onto the Illumina MiSeq cartridge using MiSeq Reagent Kit v3 (2x300 bp Paired-end reads, 15 Gb output) according to manufacturer instructions. Sequences were processed using FROGS [7]. Raw sequencing data quality was checked using FastQC and reads with a Phred quality score <30 were discarded. Chimeras and singletons were removed from the dataset. Quality control retained sequences with a length between 100 and 400 bp. Paired-end reads were merged using Vsearch. 16S rRNA Operational Taxonomic Units (OTUs) were assigned based on at least 99% sequence similarity to the lowest possible taxonomic rank against the SILVA Pintail 100-138 reference database.

Alpha diversity. Alpha diversity was calculated with Chao1, Shannon and Inverse Simpson indexes and compared via ANOVA. Sequences were rarefied to an even depth of 19,580 sequences per sample to account for unequal sequencing depth across samples.

Linear Discriminant Analysis Effect Size (LEfSe) analysis. LEfSe analysis was performed to estimate the effect size of each differentially abundant feature [8]. For OTUs with an average abundance in all samples greater than 0.1%, abundances were normalized to the sum of the values per sample in 1 million and then subjected to linear discriminant analysis (LDA). The LDA was performed using all-against-all strategy, and OTUs showing a score higher than 2.0 were selected.

References

[1] Martinez-Lopez N, Tarabra E, Toledo M, Garcia-Macia M, Sahu S, Coletto L, *et al.* (2017). System-wide Benefits of Intermeal Fasting by Autophagy. Cell Metab, 26:856-871 e855.

[2] Folch J, Lees M, Sloane Stanley GH (1957). A simple method for the isolation and purification of total lipides from animal tissues. J Biol Chem, 226:497-509.

[3] Morrison WR, Smith LM (1964). Preparation of Fatty Acid Methyl Esters and Dimethylacetals from Lipids with Boron Fluoride--Methanol. J Lipid Res, 5:600-608.

[4] Godon JJ, Zumstein E, Dabert P, Habouzit F, Moletta R (1997). Molecular microbial diversity of an anaerobic digestor as determined by small-subunit rDNA sequence analysis. Appl Environ Microbiol, 63:2802-2813.

[5] Nadkarni MA, Martin FE, Jacques NA, Hunter N (2002). Determination of bacterial load by real-time PCR using a broad-range (universal) probe and primers set. Microbiology (Reading), 148:257-266.

[6] Lluch J, Servant F, Paisse S, Valle C, Valiere S, Kuchly C, *et al.* (2015). The Characterization of Novel Tissue Microbiota Using an Optimized 16S Metagenomic Sequencing Pipeline. PLoS One, 10:e0142334.

[7] Escudie F, Auer L, Bernard M, Mariadassou M, Cauquil L, Vidal K, *et al.* (2018). FROGS: Find, Rapidly, OTUs with Galaxy Solution. Bioinformatics, 34:1287-1294.

[8] Segata N, Izard J, Waldron L, Gevers D, Miropolsky L, Garrett WS, *et al.* (2011). Metagenomic biomarker discovery and explanation. Genome Biol, 12:R60.

© 2023. Lapaquette P et al. Published online at http://www.aginganddisease.org/EN/10.14336/AD.2023.0324

SUPPLEMENTARY DATA

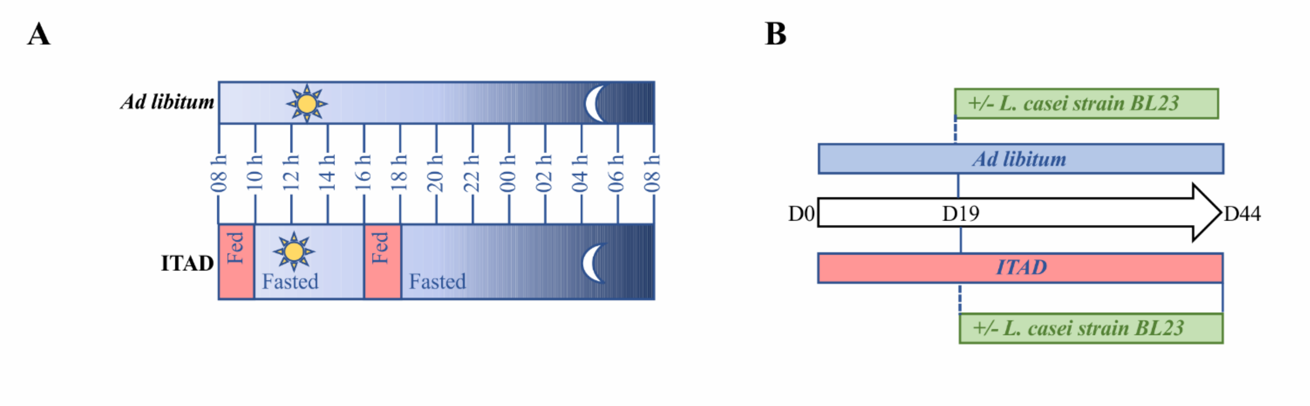

Supplementary Figure 1. Feeding and probiotic supplementation protocols. (**A**) Mice had unrestricted access to food all day and all night (*ad libitum*), or mice had access to food only from 8 am to 10 am and from 4 pm to 6 pm (isocaloric twice-a-day feeding, ITAD) for 44 days. (**B**) ITAD fed-mice and *ad libitum*-fed mice received daily either PBS buffer or *Lacticaseibacillus casei* (*L. casei*) BL23 by oral gavage during the last 25 days of the experiment. n=8 per subgroup.

© 2023. Lapaquette P et al. Published online at http://www.aginganddisease.org/EN/10.14336/AD.2023.0324
